# The Impact of Mindfulness Training on Police Officer Stress, Mental Health, and Salivary Cortisol Levels

**DOI:** 10.3389/fpsyg.2021.720753

**Published:** 2021-09-03

**Authors:** Daniel W. Grupe, Jonah L. Stoller, Carmen Alonso, Chad McGehee, Chris Smith, Jeanette A. Mumford, Melissa A. Rosenkranz, Richard J. Davidson

**Affiliations:** ^1^Center for Healthy Minds, University of Wisconsin-Madison, Madison, WI, United States; ^2^Colorado School of Public Health, University of Colorado Denver, Aurora, CO, United States; ^3^Just Mindfulness, Madison, WI, United States; ^4^Department of Athletics, University of Wisconsin-Madison, Madison, WI, United States; ^5^Academy for Mindfulness, Glendale, WI, United States; ^6^Department of Psychology, Stanford University, Stanford, CA, United States; ^7^Department of Psychology, University of Wisconsin-Madison, Madison, WI, United States; ^8^Department of Psychiatry, University of Wisconsin-Madison, Madison, WI, United States

**Keywords:** law enforcement, stress, mental health, cortisol, mindfulness, posttraumatic stress disorder, inflammation, sleep

## Abstract

Unaddressed occupational stress and trauma contribute to elevated rates of mental illness and suicide in policing, and to violent and aggressive behavior that disproportionately impacts communities of color. Emerging evidence suggests mindfulness training with police may reduce stress and aggression and improve mental health, but there is limited evidence for changes in biological outcomes or the lasting benefits of mindfulness training. We conducted a randomized controlled trial (RCT) of 114 police officers from three Midwestern U.S. law enforcement agencies. We assessed stress-related physical and mental health symptoms, blood-based inflammatory markers, and hair and salivary cortisol. Participants were then randomized to an 8-week mindfulness intervention or waitlist control (WLC), and the same assessments were repeated post-intervention and at 3-month follow-up. Relative to waitlist control, the mindfulness group had greater improvements in psychological distress, mental health symptoms, and sleep quality post-training, gains that were maintained at 3-month follow-up. Intervention participants also had a significantly lower cortisol awakening response (CAR) at 3-month follow-up relative to waitlist control. Contrary to hypotheses, there were no intervention effects on hair cortisol, diurnal cortisol slope, or inflammatory markers. In summary, an 8-week mindfulness intervention for police officers led to self-reported improvements in distress, mental health, and sleep, and a lower CAR. These benefits persisted (or emerged) at 3-month follow-up, suggesting that this training may buffer against the long-term consequences of chronic stress. Future research should assess the persistence of these benefits over a longer period while expanding the scope of outcomes to consider the broader community of mindfulness training for police.

Clinical Trial Registration: ClinicalTrials.gov#NCT03488875.

## Introduction

There is a pressing need for evidence-based interventions to address twin crises of police officer mental health and the violent and discriminatory treatment of communities of color and marginalized groups by police. Daily exposure to direct and vicarious trauma, chronic organizational stressors, and heightened police-community tension contribute to elevated rates of posttraumatic stress, depression, alcoholism, and suicide in police officers relative to the general public (Ballenger et al., 2011; Carleton et al., 2017; Violanti et al., 2017a; Chopko et al., 2018; Syed et al., 2020). The fatigue, burnout, and hypervigilance that emerge in the absence of effective emotion regulatory strategies contribute to aggressive and discriminatory policing practices (Kop et al., 1999; Rajaratnam et al., 2011; Ma et al., 2013; Goff and Rau, 2020), exacerbating distrust and anger toward the police, particularly among communities of color that historically have been disproportionately impacted.

Recent research suggests mindfulness-based interventions (MBIs) may help address each of these crises. Commonly conceptualized as providing “stress reduction” techniques, the embodied practices and didactic knowledge contained within MBIs enhance self-awareness and self-regulation (Vago and Silbersweig, 2012), deepen one’s sense of interconnection and compassion (Hutcherson et al., 2008; Kang et al., 2014), and encourage exploration and acceptance of challenging emotions (Thompson and Waltz, 2010; Lindsay and Creswell, 2017) as an alternative to culturally engrained patterns of avoidance and emotional control (Pogrebin and Poole, 1995; Karaffa and Tochkov, 2013). Two pilot studies demonstrated the acceptability, feasibility, and preliminary efficacy of MBIs adapted for police personnel (Christopher et al., 2016; Grupe et al., 2021a), and three randomized controlled trials (RCTs) have demonstrated benefits of mindfulness training relative to waitlist control (WLC) for self-reported stress, burnout, mindfulness, alcohol use, negative affect, and global health (Christopher et al., 2018; Krick and Felfe, 2019; Trombka et al., 2021). Results for anxiety and depression symptoms have been mixed, which could partially be attributable to differences in sample characteristics: a study of primarily male police officers in the U.S. Northwest found no differences (Christopher et al., 2018), whereas a study of primarily female officers in Brazil found sizable and durable reductions in anxiety and depression symptoms (Trombka et al., 2021).

Several gaps remain in our knowledge of the benefits of mindfulness training for police officer health and well-being. First, data on stress-related biological outcomes, particularly biomarkers related to cortisol and inflammation, are limited. Prolonged activation of the hypothalamic-pituitary-adrenal (HPA) axis and excessive cortisol release contribute to widespread dysregulation of central and peripheral biological systems influenced by this hormone (McEwen, 1998). Among other deleterious consequences, prolonged HPA axis activation lessens cortisol’s ability to suppress inflammatory responses (Rohleder, 2012). Elevated inflammation is consequently associated with posttraumatic stress disorder (PTSD), depression, cardiovascular disease, and metabolic syndrome (Raison et al., 2006; Eraly et al., 2014; Lindqvist et al., 2014; Liu et al., 2017). Notably, MBIs have demonstrated benefits for immune system function, including relatively consistent decreases in C-reactive protein (CRP; Black and Slavich, 2016). No studies to our knowledge have investigated the impact of mindfulness training on inflammation in police officers, although one study provided tentative evidence for a lower cortisol awakening response (CAR) following mindfulness training (Christopher et al., 2018).

Second, data on the long-term impact of MBIs for police are also limited and inconsistent. One RCT in 61 United States police officers failed to find group differences 3 month post-training (Christopher et al., 2018) whereas another RCT in 170 Brazilian police officers reported robust benefits for mindfulness training vs. waitlist at 6-month follow-up for quality of life, anxiety, and depression (Trombka et al., 2021). The latter study is consistent with a single-arm pilot study that identified reductions in burnout, anxiety, and PTSD symptoms at 5-month follow-up (Grupe et al., 2021a). Demonstrating evidence for long-term benefits of these interventions is critical both for police officer well-being and that of the communities they serve.

We conducted a RCT of an 8-week adapted mindfulness training vs. waitlist control in 114 police officers from a medium-sized Midwestern United States city, recruiting from a city police department, university police department, and a county sheriff’s agency. In addition to self-reported stress and mental/physical health, we collected data on biomarkers of cortisol release and inflammation immediately post-training and at 3-month follow-up. The focus of this report is on self-reported stress, stress-related physical and mental health, and biomarkers of relevance for stress-related illness. In the interest of coherence and brevity, additional outcomes are described briefly below but results will be reported elsewhere.

## Materials and Methods

### Participants and Recruitment

Participants were sworn law enforcement officers employed by the Dane County Sheriff’s Office, Madison Police Department, and University of Wisconsin-Madison Police Department. Participants were enrolled in two discrete cohorts, with 60 participants enrolled in Cohort 1 (March–April 2018) and 55 in Cohort 2 (February–March 2019). Participants self-selected into the study and participation, while encouraged by participating agencies, was fully voluntary. There were no inclusion criteria that participants needed to meet, for example with regard to pre-existing symptomology or health concerns. Exclusionary criteria included significant previous meditation practice or participation in a previous mindfulness class. For the first cohort of participants, personnel in command positions (lieutenant or above) were excluded, as were sheriff’s deputies working primarily in the county jail; these individuals were eligible for the second cohort.

**Figure 1:**
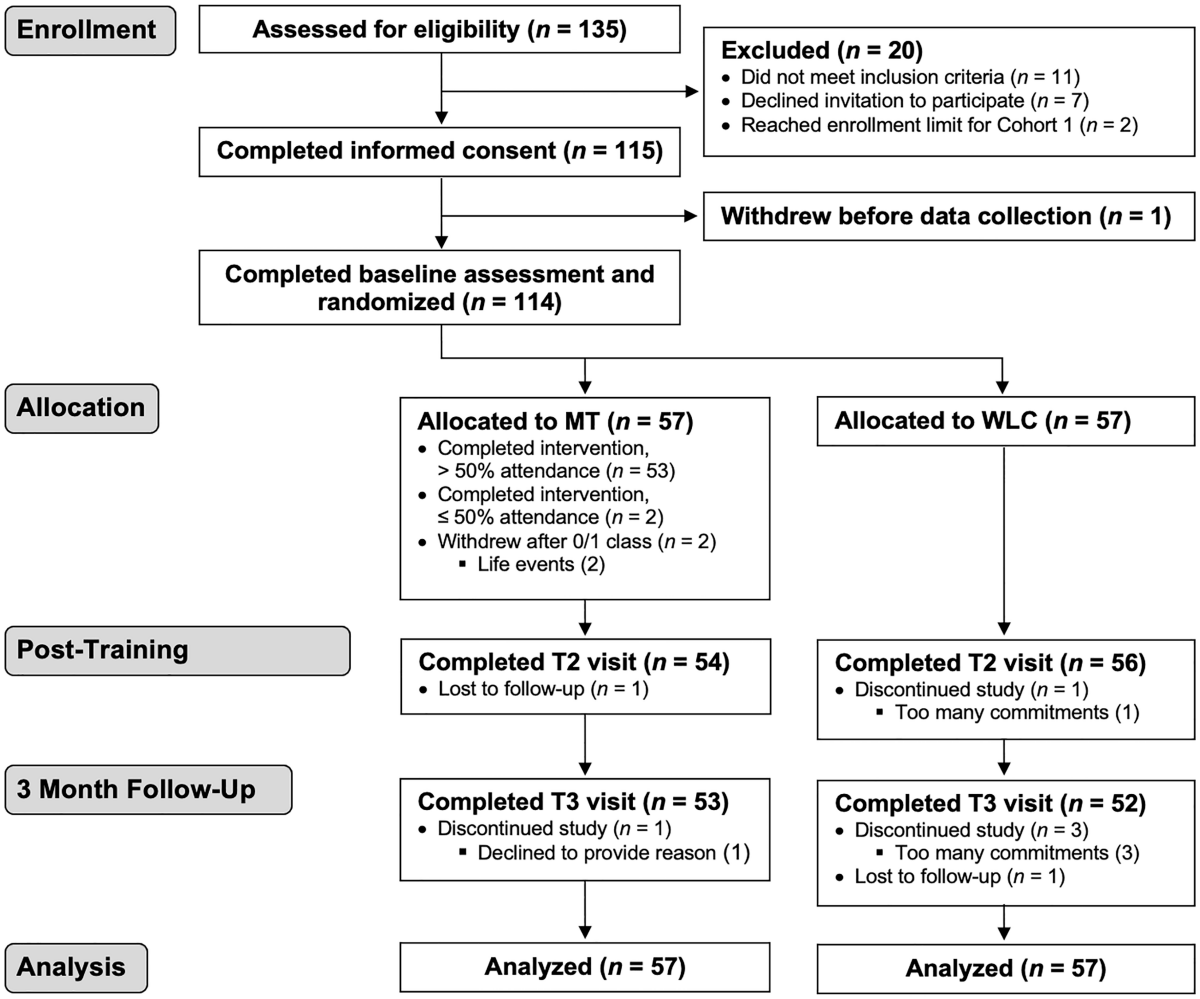
CONSORT diagram. Primary intent-to-treat analysis includes all participants with baseline data collection (*n*=114). MT, mindfulness training and WLC, waitlist control.

Prior to recruitment, we informally advertised the study to an officer advisory council, union leadership, and wellness committee members. Prior to Cohort 2, we spoke with key department stakeholders to develop strategies for engaging officers of color in this research, which resulted in a more racially and ethnically representative cohort of participants. Formal recruitment activities included emails sent by agency liaisons, flyers posted at district stations, and announcements at daily briefings. Interested individuals contacted our study team and completed a phone screening to confirm eligibility (see Figure 1 for CONSORT diagram).

### Data Collection

#### In-Person Data Collection

Informed consent and in-person baseline assessments took place on average 15.4days before randomization (range=3–29). The same assessments occurred post-training (average 12.1days, range=3–26) and at 3-month follow-up (average 94.2days after post-training assessment, range=71–116). The two groups did not differ in the average amount of time between the intervention and post-test [mindfulness training=12.3days, waitlist control=11.9days; *t*(107)=0.38, *p*=0.71]. The follow-up assessment took place on average 3days later for the mindfulness group (107.5days) relative to waitlist control [104.6days; *t*(102)=2.04, *p*=0.04].

At each visit, we collected accuracy and reaction time data on two computerized behavioral tasks, the mnemonic similarity task (Stark et al., 2013), and the affective go/no-go task (Hare et al., 2005; results not presented here). We obtained capillary blood on filter paper following lancet prick to assess inflammatory markers from dried blood spots (DBSs), and collected hair samples from 1″ below the vertex of participants’ scalps for hair cortisol concentration measurement. Participants completed a battery of self-report measures (Table 2; Supplementary Table S2) and a work and demographics questionnaire.

#### Field Data Collection

Following the in-person visit, participants collected field data over the course of their next scheduled work week. Participants collected four saliva samples using Salivette® cortisol tubes (Sarstedt, Nümbrecht) on each of 3 consecutive days – immediately after awakening, 30 and 45min after awakening, and immediately before bed – and indicated the time of collection on paper logs. Participants were instructed to avoid eating, drinking, brushing teeth, smoking, or exercising prior to saliva collection. Participants were provided with Fitbit Charge 2 activity trackers to assess heart rate and sleep throughout the week (analysis is ongoing; see https://osf.io/mqnga). At the end of each workday, participants completed an in-house “work events log” asking about exposure and perceived stress for work stressors (Chen and Grupe, under review). At the conclusion of the week, participants returned data collection materials in a postage-paid box.

### Randomization

Block randomization to mindfulness training or waitlist control (stratified by police agency) occurred 1week before classes began. Seven participant pairs and one group of four participants were randomized within “mini-strata,” due to overlapping job responsibilities or to avoid assigning supervisor/supervisee pairs to the same class.

### Intervention

The intervention was an 8-week, 18-h mindfulness-based training lightly modified from a previous feasibility pilot study (Grupe et al., 2021a,b). This police-specific training, inspired by Mindfulness-Based Stress Reduction (MBSR) and Mindfulness-Based Resilience Training (Christopher et al., 2016), is intended to promote resilient responses to the chronic and acute stressors of policing and to support officers’ humanity and well-being. Weekly 2-h classes (and a 4-h class in week 7) consisted of didactic instruction around principles of mindfulness, stress, emotions, and scientific research on mindfulness; embodied practices, including mindfulness of the breath and body, a body scan, walking meditation, mindful movement (including adapted yoga or tai chi), mindfulness of thoughts and emotions, mindful speaking and listening, mindful eating, and compassion practice; and inquiry in dyads, triads, or the full group about participants’ experiences in practice. Adaptations from traditional MBSR trainings included slightly shorter classes to better accommodate work schedules, shorter homework assignments to encourage more frequent if lighter engagement, language and practices tailored to police work and culture (and informed by time spent by our instructors with police officers in and out of work leading up to the classes), and the inclusion of informal mindfulness practices integrated into specific aspects of police work. For a curriculum overview, see Appendix 1 [and for greater discussion of police-specific adaptations, see Grupe et al. (2021b)].

Participants were provided with guided practices recorded by instructors and police officers and were encouraged to practice 6days/week, beginning with 9min/day and eventually increasing to 20min/day. The instructors offered participants suggestions for informal mindfulness practices (e.g., “drop-ins” to present-moment experiences, brief breath manipulations, and cultivating “mindful pauses”) that they could integrate into daily activities. Participants were provided with paper and electronic logs (Appendix 2) to record formal and informal practice. Similar logs were used to track practice time between the end of the class and the follow-up assessment; because not all participants received and utilized these logs, estimates of practice time over this interval were based on retrospective practice reporting at follow-up (see “Data Analysis”).

### Data Processing

#### Self-Report

We collected 18 self-report (sub)scales related to stress and physical/mental health: the Organizational and Operational Police Stress Questionnaire (McCreary and Thompson, 2006); Perceived Stress Scale (Cohen and Williamson, 1988); PTSD Checklist for DSM-5 (Weathers et al., 2013); eight subscales from the Patient-Reported Outcomes Measurement Information System (PROMIS anxiety, depression, fatigue, sleep disturbances, ability to participate in social roles and activities, physical function, pain interference, and pain intensity); Pittsburgh Sleep Quality Inventory (Buysse et al., 1989); Alcohol Use Disorders Identification Test (AUDIT; Saunders et al., 1993); Oldenburg Burnout Inventory (Halbesleben and Demerouti, 2005); Health Behaviors Checklist (HBC; Hampson et al., 2017); and Work Limitations Questionnaire (Lerner et al., 2002). We obtained a broad array of self-report outcomes because of the eclectic nature of MBIs and the relative lack of RCTs on these interventions in law enforcement populations. In order to limit the number of comparisons and test the impact of mindfulness training on broad domains of stress and health, data-driven reduction of these (sub)scales into five discrete components was accomplished using principal component analysis (PCA) in R (see Results).

#### Biological Outcomes

##### Salivary Cortisol

Salivettes were centrifuged and samples stored at −80° until shipment to the lab of Dr. Nicolas Rohleder at Brandeis University. Salivary cortisol was measured using a commercially available luminescence immunoassay (CLIA; IBL-Hamburg, Hamburg, Germany). Intra- and inter-assay coefficients of variation were 5.61 and 8.64% (2018 cohort) and 5.32 and 8.42% (2019 cohort), respectively. Raw values were log-transformed, and observations greater than three SDs from the mean of each sampling time were excluded. The CAR (CAR) was operationalized as the difference between the greater of the 30/45-min values and the waking value. Diurnal slope was operationalized as the difference between the waking and bedtime values, divided by the number of minutes between these samples. For days with a negative CAR – suggesting non-compliance for the waking sample – both the CAR and slope were excluded (21% of observations across timepoints). We additionally excluded the 16 nightshift workers (14% of the sample) from salivary cortisol analyses as we expected these individuals would demonstrate categorically distinct diurnal responses.

##### Hair Cortisol Concentration

Hair cortisol concentration from the 3-cm segment proximal to the scalp was measured using immunoassay methods in the lab of Dr. Clemens Kirschbaum at Technische Universität Dresden. Samples with no discernable cortisol were assigned values of 0.15pg/mg (the lower detection limit). Samples with “supra-physiological” levels of cortisol (operationalized as 3SD above the mean) were assumed to be contaminated (Wang et al., 2019) and were excluded (1% of samples). Log-transformed values were used for analysis.

##### Inflammatory Markers

Dried blood spot samples were stored at −80° until shipment to the lab of Dr. Thom McDade at Northwestern University. CRP was quantified using an updated version of a protocol previously validated for use with DBS samples (Schmid et al., 2004). CRP values >10mg/ml were assumed to reflect acute infection and were removed (1% of samples). The inflammatory cytokines interleukin (IL)-6, IL-10, and tumor necrosis factor (TNF)-α were quantified as described in a recent validation study (McDade et al., 2020). All inflammatory markers were log-transformed, and a summary measure of the three cytokines was derived by taking the mean of Z-transformed scores at each timepoint (normalized to mean baseline values).

### Data Analysis

Statistical analyses were conducted in RStudio (Version 1.2.5042; RStudio Team, 2020) in the R programming environment (Version 3.6.3; R Core Team, 2020).

Using the “lmerTest” library, we conducted intent-to-treat, linear mixed effects analyses, including all observations at all timepoints. Models included baseline scores for each outcome as a fixed covariate (for salivary cortisol analyses, the within-subject average across baseline sampling days), a random intercept, and fixed covariates of gender, years of police experience, and cohort. Cortisol and DBS analyses additionally controlled for BMI; cortisol analyses controlled for smoking status; hair cortisol analyses controlled for hair dye or similar treatments; and salivary cortisol analyses controlled for workday. We were unable to model the non-independence of participants within mindfulness classes, as the small number of classes led to convergence errors.

The significance of Group*Time interactions (across post-intervention and follow-up timepoints) was assessed using the anova.lmerModLmerTest() function to compare models with and without the interaction term. We also calculated group differences at post-intervention and follow-up timepoints individually. We tested for “dose-response” effects by correlating change scores for each outcome with self-reported practice minutes during the class. For the follow-up period, we used retrospective self-report to classify participants as “high engagement” (≥2days of formal practice/week; *N*=22) or “low engagement” (≤1day of formal practice/week; *N*=32) and compared these groups on change scores over the same period.

### Pre-registration

Salivary and hair cortisol processing and analysis followed our Open Science Framework pre-registration^1^ with two exceptions. First, we adjusted for baseline DV values to address regression to the mean. Second, we removed the random slope and nesting for mixed models to remedy convergence errors (Matuschek et al., 2017). We only report here results for intervention effects (Hypotheses 1b, 2b, and 3b). We did not pre-register hypotheses for other outcomes.

## Results

### Participants Flow and Intervention Engagement

One participant withdraw following consent and prior to data collection, leaving 114 participants for intent-to-treat analyses (Figure 1). Information on demographics and job characteristics is in Table 1.

**Table 1:** Demographic and work information.

Characteristic	MT	WLC
	*N* (%)	*N* (%)
**Gender**		
Female	23 (40)	24 (42)
Male	34 (60)	33 (58)
**Race**		
Caucasian	46 (81)	49 (86)
Black/African American	2 (4)	2 (4)
Asian	3 (5)	0
American Indian/Alaskan Native	0	2 (4)
More than one race	4 (7)	4 (7)
Unknown	2 (4)	0
**Ethnicity**		
Hispanic or Latinx	1 (2)	2 (4)
Not Hispanic or Latinx	55 (96)	55 (96)
Unknown	1 (2)	0
**Marital Status** ^a^		
Married	16 (59)	17 (63)
Unmarried relationship	6 (22)	6 (22)
Divorced	2 (7)	2 (7)
Unknown	3 (11)	2 (7)
**Education**		
Some college education	4 (7)	9 (16)
Four-year college degree	38 (67)	34 (60)
Some post-graduate education	4 (7)	7 (12)
Post-graduate/professional degree	11 (19)	7 (12)
**Agency**		
Madison Police Department	33 (58)	31 (54)
Dane County Sheriff’s Office	19 (33)	21 (37)
UW-Madison Police Department	5 (9)	5 (9)
**Daily work schedule**		
1st detail (~0600–1400)	28 (49)	27 (47)
2nd detail (~1200–2000)	4 (7)	4 (7)
3rd detail (~1400–2200)	18 (32)	17 (30)
4th detail (~2000–0400)	4 (7)	2 (4)
5th detail (~2200–0600)	3 (5)	7 (12)
**Rank/job responsibilities**		
Captain	1 (2)	0
Lieutenant	1 (2)	2 (4)
Sergeant	4 (7)	7 (12)
Detective	12 (21)	9 (16)
Investigator	1 (2)	2 (4)
Police Officer	25 (44)	20 (35)
Sheriff’s Deputy	13 (23)	17 (30)
	**Mean (SD)**	**Mean (SD)**
Age	40.2 (7.4)	39.8 (9.3)
Years police experience	13.8 (7.9)	14.3 (8.3)

*Due to rounding, percentages may not total 100%. MT, mindfulness training; WLC, waitlist control*.

a *Information on marital status was collected for participants in Cohort 2 only (n=27 for each group)*.

Intervention engagement was excellent, with 55/57 participants completing the mindfulness training. Overall class attendance was 80.3%, with 53/57 participants attending more than 50% of the classes. Participants reported a median of 42days of formal meditation practice over 8weeks (range=0–55), and a median of 130 weekly practice minutes (8-week range=0–2,669; Supplementary Figure S1).

### Principal Component Analysis of Self-Report Data

We used PCA to reduce 18 self-report (sub)scales of stress, mental health, and physical health into five components with an eigenvalue cut-off of 1.0. After reviewing item loadings, we sequentially removed Oldenburg-disengagement and AUDIT scores, which each loaded <0.40 on all components. The remaining items each loaded >0.60 on a single component, and we retained this 16-item, 5-component structure for subsequent analyses (Supplementary Table S1). These five components reflected psychological distress/mental health (Component 1, eight items, and 28% total variance explained), pain (Component 2, two items, and 11% variance), physical health (Component 3, two items, and 10% variance), occupational stress (Component 4, two items, and 12% variance), and sleep disturbances (Component 5, two items, and 13% variance).

### Effects of Mindfulness Training on Self-Reported Stress and Health Outcomes

**Figure 2:**
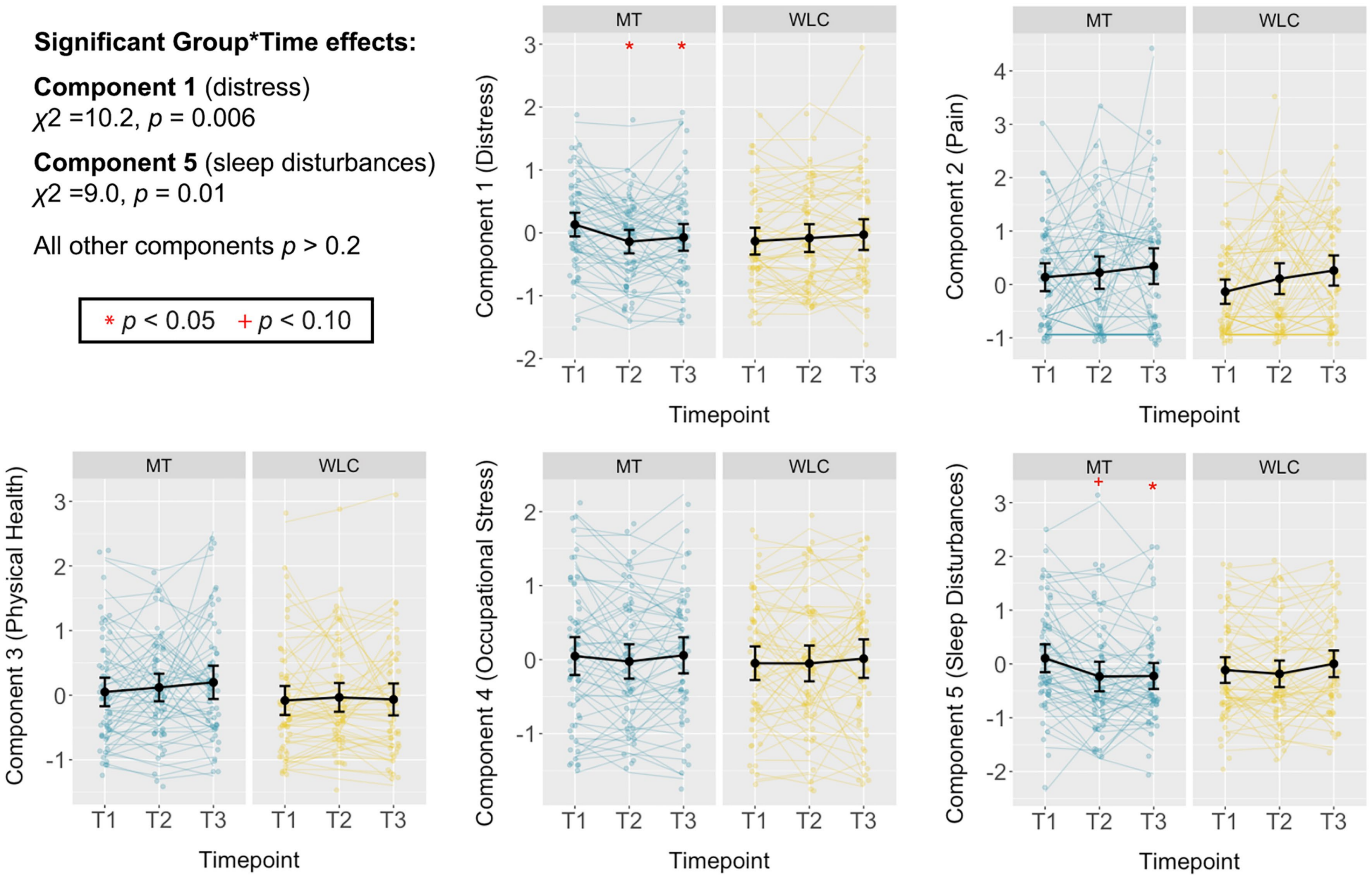
Effects of mindfulness training on self-reported, stress-relevant health outcomes. Standardized scores for each self-report domain identified through principal component analysis (PCA), plotted separately for mindfulness training (MT), and waitlist control (WLC) groups at baseline (T1), post-training (T2), and 3-month follow-up (T3). Relative to WLC, participants in the MT group had a significant reduction in self-report outcomes related to psychological distress and mental health symptoms (Component 1; *χ*^2^=10.2, *p*=0.006), with group differences evident at T2 and T3 when controlling for scores at T1. A significant Group*Time interaction was also seen for sleep disruptions (Component 5; *χ*^2^=9.0, *p*=0.01), although this difference was significant at T3 only. No other intervention effects were seen (*p*s>0.2). Summary statistics are within-group means with 95% CIs.

A significant Group*Time interaction for Component 1 reflected reduced distress and mental health symptoms following mindfulness training relative to waitlist control, controlling for baseline symptoms, cohort, gender, and years of policing [*χ*^2^(2, *N*=10)=10.21, *p*=0.006, η^2^=0.09; Figure 2; Table 2]. Improved mental health and reduced distress were observed immediately after the 8-week training [*t*(172.9)=−2.96, *p*=0.003, *d*=−0.45] and at 3-month follow-up [*t*(176.9)=−2.69, *p*=0.008, *d*=−0.41]. Examination of individual subscales showed significant Group*Time interactions (uncorrected for multiple comparisons) for the PTSD Checklist, PROMIS-anxiety, PROMIS-fatigue, and Oldenburg-exhaustion, with marginal interactions (and significant group differences at 3-month follow-up only) for the Perceived Stress Scale and PROMIS-depression (see Table 2 for test statistics, including corrected/uncorrected *p* values).

**Table 2:** Differences between mindfulness training and waitlist control groups for all outcomes.

	Omnibus group*time				Group differences at time 2					Group differences at time 3				
Measure	*χ* ^2^	*p*	*p*_fdr_	η^2^	*t*	df	*p*	*p*_fdr_	*d*	*t*	df	*p*	*p*_fdr_	*d*
Component 1: distress	10.21	0.006	-	0.09	−2.96	172.9	0.003	-	−0.45	−2.69	176.9	0.008	-	−0.41
Perceived stress scale	5.68	0.06	0.09	0.05	−1.62	183.5	0.11	0.18	−0.24	−2.30	187.5	0.02	0.04	−0.34
PTSD checklist	6.20	0.04	0.09	0.05	−2.52	163.2	0.01	0.04	−0.40	−1.44	168.1	0.15	0.20	−0.22
PROMIS anxiety	7.08	0.03	0.09	0.06	−2.27	176.7	0.02	0.05	−0.34	−2.37	180.1	0.02	0.04	−0.35
PROMIS depression	5.42	0.07	0.09	0.05	−1.47	168.6	0.14	0.19	−0.23	−2.34	173.6	0.02	0.04	−0.36
PROMIS fatigue	5.98	0.05	0.09	0.05	−2.01	185.0	0.05	0.10	−0.30	−2.14	188.2	0.03	0.05	−0.31
PROMIS social participation	0.40	0.82	0.82	0.00	−0.22	178.5	0.82	0.82	−0.03	0.43	182.3	0.67	0.73	0.06
Work limitations questionnaire	0.91	0.63	0.72	0.01	-0.81	207.4	0.42	0.48	−0.11	0.35	208.4	0.73	0.73	0.05
OLBI exhaustion	8.28	0.02	0.09	0.07	−2.66	177.8	0.008	0.04	−0.40	−2.31	181.5	0.02	0.04	−0.35
Component 2: pain	0.03	0.99	-	0.00	0.07	193.1	0.95	-	0.01	−0.12	195.7	0.90	-	−0.02
PROMIS pain intensity	0.00	1.00	1.00	0.00	−0.01	188.2	0.99	0.99	0.00	0.03	191.7	0.98	0.98	−0.00
PROMIS pain interference	0.06	0.97	1.00	0.00	0.21	197.1	0.83	0.99	0.03	−0.04	199.3	0.97	0.98	−0.01
Component 3: physical health	2.82	0.24	-	0.01	0.32	214.9	0.75	-	0.05	1.67	215.0	0.10	-	0.23
Health behavior checklist	1.25	0.53	0.53	0.01	0.89	190.2	0.38	0.39	0.13	−0.34	194.0	0.74	0.74	−0.05
PROMIS physical function	2.37	0.31	0.53	0.02	0.86	207.2	0.39	0.39	0.12	1.42	208.2	0.16	0.32	0.20
Component 4: police stress	0.45	0.80	-	0.00	−0.66	171.5	0.51	-	−0.10	−0.27	175.3	0.79	-	−0.04
Operational PSQ	0.40	0.82	0.90	0.00	−0.60	168.0	0.55	0.64	−0.09	−0.13	172.0	0.90	0.90	−0.02
Organizational PSQ	0.21	0.90	0.90	0.00	−0.46	178.0	0.64	0.64	−0.07	−0.21	181.4	0.83	0.90	−0.03
Component 5: disrupted sleep	9.01	0.01	-	0.08	−1.66	186.1	0.10	-	−0.25	−2.99	189.3	0.003	-	−0.44
Pittsburgh sleep quality index	4.13	0.13	0.13	0.04	−0.42	177.1	0.67	0.67	−0.06	−1.98	179.1	0.05	0.05	−0.30
PROMIS sleep disturbances	9.52	0.009	0.02	0.09	−2.01	197.9	0.05	0.10	−0.29	−2.87	200.1	0.005	0.01	−0.41
Cortisol awakening response (CAR)	5.55	0.06	-	0.04	−1.31	187.6	0.19	-	−0.20	−2.31	195.3	0.02	-	−0.33
Diurnal cortisol slope	1.25	0.54	-	0.01	−0.75	116.3	0.45	-	−0.14	−0.95	125.2	0.34	-	−0.17
Hair cortisol concentration	1.03	0.60	-	0.01	0.26	119.7	0.80	-	0.05	1.01	124.7	0.31	-	0.18
High-sensitivity CRP	2.05	0.36	-	0.02	0.87	161.6	0.38	-	0.14	−0.61	167.0	0.54	-	−0.09
Cytokine 3-plex	0.39	0.82	-	0.01	0.49	141.5	0.63	-	0.08	0.49	138.3	0.62	-	0.08
IL-6	1.48	0.48	0.84	0.02	0.77	147.4	0.44	0.77	0.13	1.00	146.3	0.32	0.68	0.17
IL-10	0.46	0.79	0.84	0.01	0.65	126.1	0.52	0.77	0.12	0.42	120.1	0.68	0.68	0.08
TNF-α	0.34	0.84	0.84	0.00	−0.29	138.7	0.77	0.77	−0.05	−0.55	135.1	0.58	0.68	−0.10

*Statistics are the results of linear mixed effects models adjusted for baseline scores with covariates of gender, years of police experience, and cohort (year 1/year 2), and additional covariates for biological markers as indicated in Materials and Methods, and a random intercept for each participant. p_fdr_=false discovery rate-corrected p values within each component measure. PTSD, posttraumatic stress disorder; PROMIS, Patient-Reported Outcomes Measurement Information System; OLBI, oldenburg burnout inventory; PSQ, Police Stress Questionnaire; CRP, C-reactive protein; IL, interleukin; and TNF, tumor necrosis factor*.

A significant Group*Time interaction was also observed for Component 5, reflecting improved sleep quality following mindfulness training relative to waitlist control [*χ*^2^(2, *N*=10)=9.01, *p*=0.01, η^2^=0.08; Figure 2; Table 2]. This difference was not significant immediately post-training [*t*(186.1)=−1.66, *p*=0.10, *d*=−0.25] but was significant at 3-month follow-up [*t*(189.3)=−2.99, *p*=0.003, *d*=−0.44]. Examination of individual subscales showed a significant Group*Time interaction for PROMIS-sleep disturbances but not the Pittsburgh Sleep Quality Inventory, which showed group differences at 3-month follow-up only (Table 2).

There were no significant Group*Time interactions for pain [Component 2; *χ*^2^(2, *N*=10)=0.03, *p*=0.99, η^2^=0.00], physical health [Component 3; *χ*^2^(2, *N*=10)=2.82, *p*=0.24, η^2^=0.01], or occupational stress [Component 4; *χ*^2^(2, *N*=10)=0.45, *p*=0.80, η^2^=0.00], and no group differences at post-training (|*t*s|<0.7, *p*s>0.5) or 3-month follow-up (|*t*s|<1.3, *p*s>0.2; Figure 2; Table 2).

We tested for “dose-response” effects by relating formal practice during the 8-week class with change scores over the same interval, which revealed no significant relationships with any self-report components (|*r*s|<0.24, *p*s>0.09). High-vs.-low practice engagement over the 3-month follow-up period was associated with improved physical health over this interval [*t*(52)=−2.35, *p*=0.02, *d*=−0.31]. Greater practice was also associated with a significant *increase* in occupational stress over this period [*t*(52)=2.16, *p*=0.04, *d*=0.29]. Practice engagement was not associated with changes in distress/mental health, pain, or sleep disturbances (|*t*s|<1.4, *p*s>0.1).

### Effects of Mindfulness Training on Cortisol

**Figure 3:**
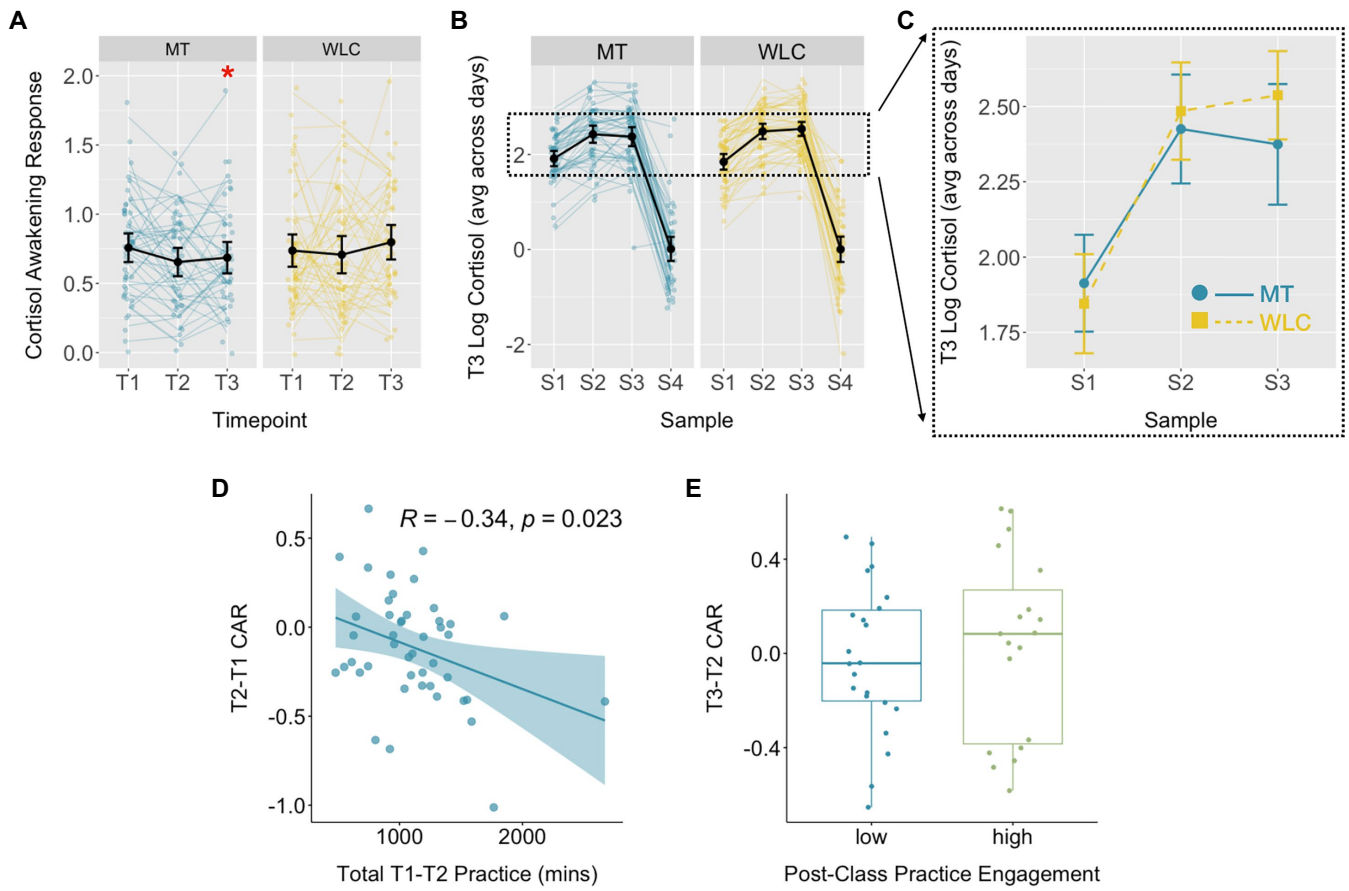
Effects of mindfulness training on the CAR. **(A)** Log-transformed CAR for the MT and WLC groups at baseline (T1), post-mindfulness training (T2), and 3-month follow-up (T3). Controlling for T1 CAR, the MT group had a significantly lower CAR at T3 relative to WLC [*t*(195.3)=2.31, *p*=0.02]. **(B,C)** Log cortisol values at each of four sampling times for T3 show a steeper rise in cortisol between S1 (awakening) and S2 (30min) for the WLC vs. MT group. Group means diverge further at S3 (45min), with the WLC group showing a slight increase and the MT group a slight decrease. **(D)** Within the MT group, greater formal mindfulness practice during the 8-week class was associated with a greater reduction in the CAR between T1 and T2. **(E)** There was no difference between groups with relatively high vs. low practice engagement between post-training and follow-up assessments and changes in the CAR over the same interval. Summary statistics are within-group means with 95% CI.

The CAR measured over 3 workdays showed a marginally significant Group*Time interaction, controlling for baseline CAR, cohort, gender, years of policing, smoking status, and BMI [*χ*^2^(2, *N*=13)=5.55, *p*=0.06, η^2^=0.04; Figure 3; Table 2]. There was no significant group difference post-training [*t*(187.6)=−1.31, *p*=0.19, *d*=−0.20], but the mindfulness group had a significantly lower CAR relative to waitlist control at follow-up [*t*(195.3)=−2.31, *p*=0.02, *d*=−0.33].

Greater practice time during the class was associated with a greater reduction in the CAR between baseline and post-training assessments [*r*(43)=−0.34, *p*=0.02; Figure 3]. CAR changes between post-training and 3-month follow-up did not differ as a function of practice engagement over this period [*t*(39)=0.49, *p*=0.63].

Group*Time interactions were not significant for diurnal cortisol slope [*χ*^2^(2, *N*=13)=1.25, *p*=0.54, η^2^=0.01] or hair cortisol concentration [*χ*^2^(2, *N*=13)=1.03, *p*=0.60, η^2^=0.01; Table 2; Supplementary Figure S2]. There were no significant relationships between formal practice time and changes in diurnal slope or hair cortisol concentration, either during the class (|*r*s|<0.22, *p*s>0.2) or over the 3-month follow-up interval (|*t*s|<1.4, *p*s>0.1).

### Effects of Mindfulness Training on Inflammatory Markers

Group*Time interactions were not significant for CRP [*χ*^2^(2, *N*=11)=2.05, *p*=0.36, η^2^=0.02], the cytokine 3-plex [*χ*^2^(2, *N*=11)=0.39, *p*=0.82, η^2^=0.02], or any individual cytokines (all *χ*^2^<1.5, all *p*s>0.4; Table 2; Supplementary Figure S3). There were no significant relationships between formal practice time and changes in inflammatory markers, either during the class (|*r*s|<0.28, *p*s>0.1) or over the 3-month follow-up interval (|*t*s|<2.0, *p*s>0.06).

## Discussion

Adding to a growing literature on the benefits of mindfulness training for police officers, we found a police-specific, 8-week MBI led to improvements in psychological distress, mental health, and subjective sleep quality, with no impact on pain, physical health, or occupational stress. These results replicate and extend previous RCTs of mindfulness training for police officers (Christopher et al., 2018; Trombka et al., 2021), while demonstrating evidence for persistent (or emergent) self-reported benefits at 3-month follow-up. A thorough investigation of the impact of mindfulness training on cortisol and inflammation revealed a reduced CAR for the mindfulness group at 3-month follow-up, with no effects for other stress-related biomarkers.

Our data-driven reduction of 18 self-report indices allowed us to test the impact of mindfulness training on five domains of stress and health. Mindfulness training led to significant reductions in psychological distress and mental health symptoms, consistent with previous RCTs demonstrating reduced anxiety and depression symptoms (Trombka et al., 2021), burnout and perceived stress (Christopher et al., 2018), and negative affect (Krick and Felfe, 2019). We provide the first evidence of reduced PTSD symptoms in an RCT of mindfulness training for police officers, consistent with findings from an earlier single-arm pilot study (Grupe et al., 2021a). This result is notable given high exposure to direct and vicarious trauma in policing, and the serious and potentially deadly consequences of unmitigated trauma exposure for police officers (Syed et al., 2020) and members of the public (Chemtob et al., 1997; Weiss et al., 2012).

We also observed a modest improvement in sleep quality with mindfulness training that was significant at 3-month follow-up (see also Christopher et al., 2018). Improved sleep may benefit both police officer health *and* community well-being. For example, North American police officers who screened positive for sleep disorders (40% of the sample) not only had increased rates of diabetes, heart disease, and depression, but were more likely to express anger at work, fall asleep while driving, and incur citizen complaints (Rajaratnam et al., 2011). Future longitudinal studies, including both laboratory and field outcomes, are needed to demonstrate whether improved mental health and sleep quality lead to persistent and cascading benefits for police and community well-being.

Mindfulness training was associated with a reduction in the CAR at 3-month follow-up, consistent with results from a previous RCT in police officers (Christopher et al., 2018). Although the CAR is generally thought of as an adaptive anticipatory response to prepare for the upcoming day (Powell and Schlotz, 2012), exaggerated responses are associated with excessive worry, burnout, and depression (Schlotz et al., 2004; Fries et al., 2009). On the other hand, an abnormally low CAR – as observed in individuals with elevated PTSD symptoms (Wessa et al., 2006; de Kloet et al., 2007), including police officers (Neylan et al., 2005) – also appears maladaptive, perhaps reflecting increased negative feedback sensitivity resulting from chronic stressor exposure (Miller et al., 2007; Violanti et al., 2017b). The modest effect of mindfulness training here suggests the possibility of a particular “endophenotype” that may be amenable to change with mindfulness training (i.e., police officers with an elevated CAR at baseline), whereas those who show baseline blunting are less likely to show training effects.

We saw no training effects for diurnal cortisol slope or hair cortisol concentration. The mixed cortisol results underscore the complex interactions between chronic stress and repeated trauma exposure, psychopathology, and time-varying changes in cortisol dynamics. A seminal meta-analysis demonstrated that acute stressor exposure leads to elevated cortisol levels, giving way to blunted responses with the passage of time (Miller et al., 2007). The same meta-analysis revealed differential effects for uncontrollable, physical, and traumatic stressors vs. those that are social in nature and potentially controllable. Our participants have been exposed to a broad array of stressors – psychosocial and physical, acute and chronic, and traumatic and mundane. Our limited knowledge regarding stressor timing, chronicity, and subjective impact is an impediment to understanding what kinds of changes following mindfulness training are possible or would be considered “beneficial.”

We also observed no effects for inflammatory markers. As with hair cortisol concentration and diurnal cortisol slope, inflammation has not been investigated in previous studies of mindfulness training for police officers. Given the relatively large sample size, these null findings are informative for investigators considering future studies in this area. A systematic review of MBI RCTs found some evidence for reductions in pro-inflammatory markers, particularly CRP, but many null results (Black and Slavich, 2016). Intervention effects may be more pronounced for individuals at elevated risk for systemic inflammation, such as older adults or those with high BMI (Villalba et al., 2019), a challenging hypothesis to test in our relatively young and physically healthy sample. It may be informative to challenge police officers with a psychosocial stressor and measure evoked inflammatory responses – and/or changes in stress hormones and physiology – to test whether mindfulness training alters dynamic biological stress responses, rather than baseline levels (Rosenkranz et al., 2013; Johnson et al., 2014), consistent with a stress buffering hypothesis (Creswell and Lindsay, 2014).

Improved distress and mental health persisted at 3-month follow-up, consistent with a previous 8-week MBI in Brazilian police officers (Trombka et al., 2021) but in contrast to a study in United States police officers (Christopher et al., 2018). In addition, sleep improvements and CAR reductions were not significant until the follow-up assessment. One consideration for sustained improvements is the degree of continued mindfulness practice post-intervention. Christopher et al. (2018) reported that only two of 24 participants endorsed *any* practice between post-training and follow-up assessments [Trombka et al. (2021) did not provide data on practice engagement]. In our study, 43/54 participants assessed at follow-up reported some amount of practice after the class, with 41% reporting 2 or more weekly days of practice. Unfortunately, our measure of ongoing practice engagement was retrospective and rather coarse. A critical direction for future work is integrating detailed, real-time, and objective practice measures to illuminate how ongoing meditation practice may undergird long-term health benefits.

## Limitations and Future Directions

One limitation of this study is that the mindfulness group was descriptively “less healthy” than the control group on several self-report outcomes at baseline. Although analyses controlled for baseline scores, which provides better protection against regression to the mean than difference scores (Vickers and Altman, 2001), apparent intervention effects may partially reflect random pre-intervention differences (while a common practice, testing for significant baseline differences in an RCT is non-meaningful as any differences by definition occurred by chance; de Boer et al., 2015). In support of our causal attribution of differences to the intervention, not all measures showing baseline differences resulted in post-training improvements. For example, both groups showed steady increases in pain through follow-up, and for physical health baseline differences became *more* pronounced over time. This could reflect greater body awareness following mindfulness training, although this relative worsening was not statistically significant.

Because we wanted to conduct in-person assessments during participants’ non-work days – and due to staffing limitations, cancellations, and other pragmatic considerations – the time window for the “immediate” post-test was up to 4weeks post-intervention, and the “3-month” follow-up was nearly 4months post-intervention for some participants. Although this range of testing times may have injected noise into intervention effects that are time-sensitive, it was unlikely to systematically bias our results. The two groups did not differ in the average amount of time between the intervention and post-test, and the follow-up assessment took place on average 3days later for the mindfulness group relative to waitlist control – a difference that should in theory favor the waitlist group, assuming intervention effects decay over time.

A more substantial limitation is the focus on data collected directly from officers, and consequent lack of evidence for the broader impact of this training. This research is predicated on the assumption that decreased police officer stress and increased well-being will have ripple effects for communities of color and marginalized individuals, whose health and safety are threatened daily by the police institution. While correlational evidence and laboratory studies provide theoretical support for this assumption (Kop et al., 1999; Rajaratnam et al., 2011; Ma et al., 2013; Andersen and Gustafsberg, 2016; Burke, 2020), without direct empirical evidence we cannot claim any community benefits for this training. Previous studies with police have shown reductions in self-reported anger and aggression following mindfulness training (Christopher et al., 2016, 2018); more compelling evidence for community benefits may involve data collected using objective metrics (e.g., administrative or body camera data on citizen interactions or use of force; Voigt et al., 2017; Wood et al., 2020), community members’ reports on interactions with police officers, or the direct measurement of stress and health outcomes from community members (Geller et al., 2014; Smith et al., 2019; Browning et al., 2021; Muentner et al., 2021).

As we expand this work to consider community impacts, it is critical that specific outcomes (and broader research agendas) intended to benefit marginalized communities are designed collaboratively with these communities (Strand et al., 2003). To the extent that this research has improved police officer health and wellness, it is precisely because we have engaged police agencies in a sustained manner, asked how this work can benefit them, and designed our training and research accordingly (Grupe et al., 2021a). To move toward the goal of greater justice for those whose treatment by policing has historically been unjust and inequitable, we must similarly engage marginalized communities throughout the research process to ensure this work is aligned with their priorities and values. Through this process, we may learn more about the limitations of orienting this training around outcomes of “officer resilience” or “stress reduction.” In collaboration with community members, police agencies, and contemplative teachers and practitioners, we can work to introduce a more explicit ethical and prosocial framework, attend to systems of injustice that do more to perpetuate violence and discrimination than the actions of individual “bad apples,” and consider the role of contemplative practices in bringing about transformative change for individuals and larger systems.

## Data Availability Statement

The raw data supporting the conclusions of this article will be made available by the authors, without undue reservation.

## Ethics Statement

The studies involving human participants were reviewed and approved by University of Wisconsin-Madison Minimal Risk IRB. The patients/participants provided their written informed consent to participate in this study.

## Author Contributions

DG, MR, and RD contributed to study conception and design. DG supervised all aspects of the project and wrote the first draft of the manuscript. CA, CM, and CS developed the intervention and wrote sections of the manuscript. DG and JS obtained the data. DG, JS, JM, and MR performed data analysis. All authors contributed to the article and approved the submitted version.

## Conflict of Interest

RD is the founder, president, and serves on the board of directors for the non-profit organization, Healthy Minds Innovations, Inc. CS was employed by The Academy for Mindfulness, Glendale WI, at the time of this work.

The remaining authors declare that the research was conducted in the absence of any commercial or financial relationships that could be construed as a potential conflict of interest.

## Publisher’s Note

All claims expressed in this article are solely those of the authors and do not necessarily represent those of their affiliated organizations, or those of the publisher, the editors and the reviewers. Any product that may be evaluated in this article, or claim that may be made by its manufacturer, is not guaranteed or endorsed by the publisher.

## Footnotes

^1^ https://osf.io/49px2

## References

[ref1] Andersen J. P. Gustafsberg H. (2016). A training method to improve police use of force decision making: a randomized controlled trial. SAGE Open 6, 1–13. 10.1177/2158244016638708

[ref2] Ballenger J. F. Best S. R. Metzler T. J. Wasserman D. A. Mohr D. C. Liberman A.. (2011). Patterns and predictors of alcohol use in male and female urban police officers. Am. J. Addict. 20, 21–29. 10.1111/j.1521-0391.2010.00092.x, PMID: 21175917 PMC3592498

[ref3] Black D. S. Slavich G. M. (2016). Mindfulness meditation and the immune system: a systematic review of randomized controlled trials. Ann. N. Y. Acad. Sci. 1373, 13–24. 10.1111/nyas.12998, PMID: 26799456 PMC4940234

[ref4] Browning C. R. Tarrence J. LaPlant E. Boettner B. Schmeer K. K. Calder C. A.. (2021). Exposure to police-related deaths and physiological stress among urban black youth. Psychoneuroendocrinology 125:104884. 10.1016/j.psyneuen.2020.104884, PMID: 33453595 PMC7904570

[ref5] Burke K. C. (2020). Democratic policing and officer well-being. Front. Psychol. 11:874. 10.3389/fpsyg.2020.00874, PMID: 32528350 PMC7264487

[ref6] Buysse D. J. Reynolds C. F. Monk T. H. Berman S. R. Kupfer D. J. (1989). The Pittsburgh sleep quality index: a new instrument for psychiatric practice and research. Psychiatry Res. 28, 193–213. 10.1016/0165-1781(89)90047-4, PMID: 2748771

[ref7] Carleton R. N. Afifi T. O. Turner S. Taillieu T. Duranceau S. LeBouthillier D. M.. (2017). Mental disorder symptoms among public safety personnel in Canada. Can. J. Psychiatr. 63, 54–64. 10.1177/0706743717723825, PMID: 28845686 PMC5788123

[ref8] Chemtob C. M. Novaco R. W. Hamada R. S. Gross D. M. Smith G. (1997). Anger regulation deficits in combat-related posttraumatic stress disorder. J. Trauma. Stress. 10, 17–36. 10.1002/jts.2490100104, PMID: 9018675

[ref9] Chopko B. A. Palmieri P. A. Adams R. E. (2018). Trauma-related sleep problems and associated health outcomes in police officers: a path analysis. J. Interpers. Violence 36, NP2725–NP2748. 10.1177/0886260518767912, PMID: 29642766

[ref10] Christopher M. S. Goerling R. J. Rogers B. S. Hunsinger M. Baron G. Bergman A. L.. (2016). A pilot study evaluating the effectiveness of a mindfulness-based intervention on cortisol awakening response and health outcomes among law enforcement officers. J. Police Crim. Psychol. 31, 15–28. 10.1007/s11896-015-9161-x

[ref11] Christopher M. S. Hunsinger M. Goerling R. J. Bowen S. Rogers B. S. Gross C. R.. (2018). Mindfulness-based resilience training to reduce health risk, stress reactivity, and aggression among law enforcement officers: a feasibility and preliminary efficacy trial. Psychiatry Res. 264, 104–115. 10.1016/j.psychres.2018.03.059, PMID: 29627695 PMC6226556

[ref12] Cohen S. Williamson G. (1988). “Perceived stress in a probability sample of the United States,” in The Social Psychology of Health. eds. Spacapan S. Oskamp S. (Newbury Park, CA: Sage Publications, Inc.), 31–67.

[ref13] Creswell J. D. Lindsay E. K. (2014). How does mindfulness training affect health? A mindfulness stress buffering account. Curr. Dir. Psychol. Sci. 23, 401–407. 10.1177/0963721414547415

[ref14] de Boer M. R. Waterlander W. E. Kuijper L. D. J. Steenhuis I. H. M. Twisk J. W. R. (2015). Testing for baseline differences in randomized controlled trials: an unhealthy research behavior that is hard to eradicate. Int. J. Behav. Nutr. Phys. Act. 12, 1–8. 10.1186/s12966-015-0162-z, PMID: 25616598 PMC4310023

[ref15] de Kloet C. S. Vermetten E. Heijnen C. J. Geuze E. Lentjes E. G. W. M. Westenberg H. G. M. (2007). Enhanced cortisol suppression in response to dexamethasone administration in traumatized veterans with and without posttraumatic stress disorder. Psychoneuroendocrinology 32, 215–226. 10.1016/j.psyneuen.2006.12.009, PMID: 17296270

[ref16] Eraly S. A. Nievergelt C. M. Maihofer A. X. Barkauskas D. A. Biswas N. Agorastos A.. (2014). Assessment of plasma C-reactive protein as a biomarker of posttraumatic stress disorder risk. JAMA Psychiat. 71, 423–431. 10.1001/jamapsychiatry.2013.4374, PMID: 24576974 PMC4032578

[ref17] Fries E. Dettenborn L. Kirschbaum C. (2009). The cortisol awakening response (CAR): facts and future directions. Int. J. Psychophysiol. 72, 67–73. 10.1016/j.ijpsycho.2008.03.014, PMID: 18854200

[ref18] Geller A. Fagan J. Tyler T. Link B. G. (2014). Aggressive policing and the mental health of Young urban men. Am. J. Public Health 104, 2321–2327. 10.2105/AJPH.2014.302046, PMID: 25322310 PMC4232139

[ref19] Goff P. A. Rau H. (2020). Predicting bad policing: theorizing burdensome and racially disparate policing through the lenses of social psychology and routine activities. Ann. Am. Acad. Pol. Soc. Sci. 687, 67–88. 10.1177/0002716220901349

[ref20] Grupe D. W. McGehee C. Smith C. Francis A. D. Mumford J. A. Davidson R. J. (2021a). Mindfulness training reduces PTSD symptoms and improves stress-related health outcomes in police officers. J. Police Crim. Psychol. 36, 72–85. 10.1007/s11896-019-09351-4, PMID: 33737763 PMC7963215

[ref21] Grupe D. W. Smith C. McGehee C. (2021b). “Introducing mindfulness training and research into policing: strategies for successful implementation,” in Interventions, Training, and Technologies for Improved Police Well-Being and Performance. eds. Arble E. Arnetz B. B. (IGI Global).

[ref22] Halbesleben J. R. B. Demerouti E. (2005). The construct validity of an alternative measure of burnout: investigating the English translation of the oldenburg burnout inventory. Work Stress. 19, 208–220. 10.1080/02678370500340728

[ref23] Hampson S. E. Edmonds G. W. Goldberg L. R. (2017). The health behavior checklist: factor structure in community samples and validity of a revised good health practices scale. J. Health Psychol. 24, 1103–1109. 10.1177/1359105316687629, PMID: 28810378 PMC5561507

[ref002] Hare T. A. Tottenham N. Davidson M. C. Glover G. H. Casey B. J. (2005). Contributions of amygdala and striatal activity in emotion regulation. Biol. Psychiatry 57, 624–632. 10.1016/j.biopsych.2004.12.038, PMID: 15780849

[ref24] Hutcherson C. A. Seppala E. M. Gross J. J. (2008). Loving-kindness meditation increases social connectedness. Emotion 8, 720–724. 10.1037/a0013237, PMID: 18837623

[ref25] Johnson D. C. Thom N. J. Stanley E. A. Haase L. Simmons A. N. Shih P. B.. (2014). Modifying resilience mechanisms in at-risk individuals: a controlled study of mindfulness training in marines preparing for deployment. Am. J. Psychiatr. 171, 844–853. 10.1176/appi.ajp.2014.13040502, PMID: 24832476 PMC4458258

[ref26] Kang Y. Gray J. R. Dovidio J. F. (2014). The nondiscriminating heart: lovingkindness meditation training decreases implicit intergroup bias. J. Exp. Psychol. Gen. 143, 1306–1313. 10.1037/a0034150, PMID: 23957283

[ref27] Karaffa K. M. Tochkov K. (2013). Attitudes toward seeking mental health treatment among law enforcement officers. Appl. Psychol. Crim. Justice 9, 75–99.

[ref28] Kop N. Euwema M. Schaufeli W. (1999). Burnout, job stress and violent behaviour among Dutch police officers. Work Stress. 13, 326–340. 10.1080/02678379950019789

[ref29] Krick A. Felfe J. (2019). Who benefits from mindfulness? The moderating role of personality and social norms for the effectiveness on psychological and physiological outcomes among police officers. J. Occup. Health Psychol. 25, 99–112. 10.1037/ocp0000159, PMID: 31219270

[ref30] Lerner D. Amick B. Rogers W. Malspeis S. Bungay K. Cynn D. (2002). The work limitations questionnaire 1. Med. Care 39, 72–85. 10.1097/00005650-200101000-00009 11176545

[ref31] Lindqvist D. Wolkowitz O. M. Mellon S. Yehuda R. Flory J. D. Henn-Haase C.. (2014). Proinflammatory milieu in combat-related PTSD is independent of depression and early life stress. Brain Behav. Immun. 42, 81–88. 10.1016/j.bbi.2014.06.003, PMID: 24929195

[ref32] Lindsay E. K. Creswell J. D. (2017). Mechanisms of mindfulness training: monitor and acceptance theory (MAT). Clin. Psychol. Rev. 51, 48–59. 10.1016/j.cpr.2016.10.011, PMID: 27835764 PMC5195874

[ref33] Liu Y. Z. Wang Y. X. Jiang C. L. (2017). Inflammation: the common pathway of stress-related diseases. Front. Hum. Neurosci. 11:316. 10.3389/fnhum.2017.00316, PMID: 28676747 PMC5476783

[ref34] Ma D. S. Correll J. Wittenbrink B. Bar-Anan Y. Sriram N. Nosek B. A. (2013). When fatigue turns deadly: the association Between fatigue and racial bias in the decision to shoot. Basic Appl. Soc. Psychol. 35, 515–524. 10.1080/01973533.2013.840630

[ref35] Matuschek H. Kliegl R. Vasishth S. Baayen H. Bates D. (2017). Balancing type I error and power in linear mixed models. J. Mem. Lang. 94, 305–315. 10.1016/j.jml.2017.01.001

[ref36] McCreary D. R. Thompson M. M. (2006). Development of two reliable and valid measures of stressors in policing: the operational and organizational police stress questionnaires. Int. J. Stress. Manag. 13, 494–518. 10.1037/1072-5245.13.4.494

[ref37] McDade T. W. Miller A. Tran T. T. Borders A. E. B. Miller G. (2020). A highly sensitive multiplex immunoassay for inflammatory cytokines in dried blood spots. Am. J. Hum. Biol. e23558. 10.1002/ajhb.23558. PMID: [Epub ahead of print] 33382166

[ref38] McEwen B. (1998). Protective and damaging effects of stress mediators. N. Engl. J. Med. 338, 171–179. 10.1056/NEJM199801153380307, PMID: 9428819

[ref39] Miller G. E. Chen E. Zhou E. S. (2007). If it goes up, must it come down? Chronic stress and the hypothalamic-pituitary-adrenocortical axis in humans. Psychol. Bull. 133, 25–45. 10.1037/0033-2909.133.1.25, PMID: 17201569

[ref40] Muentner L. Kapoor A. Weymouth L. Poehlmann-Tynan J. (2021). Getting under the skin: physiological stress and witnessing paternal arrest in young children with incarcerated fathers. Dev. Psychobiol. 63, 1568–1582. 10.1002/dev.22113, PMID: 33634487 PMC8530104

[ref41] Neylan T. C. Brunet A. Pole N. Best S. R. Metzler T. J. Yehuda R.. (2005). PTSD symptoms predict waking salivary cortisol levels in police officers. Psychoneuroendocrinology 30, 373–381. 10.1016/j.psyneuen.2004.10.005, PMID: 15694117

[ref42] Pogrebin M. R. Poole E. D. (1995). “Emotion management: a study of police response to tragic events,” in Social Perspectives on Emotion. *Vol*. 3. eds. Flaherty M. G. Ellis C. (Elsevier Science/JAI Press), 149–168.

[ref43] Powell D. J. Schlotz W. (2012). Daily life stress and the cortisol awakening response: testing the anticipation hypothesis. PLoS One 7:e52067. 10.1371/journal.pone.0052067, PMID: 23284871 PMC3527370

[ref44] Raison C. L. Capuron L. Miller A. H. (2006). Cytokines sing the blues: inflammation and the pathogenesis of depression. Trends Immunol. 27, 24–31. 10.1016/j.it.2005.11.006, PMID: 16316783 PMC3392963

[ref45] Rajaratnam S. M. W. Barger L. K. Lockley S. W. Shea S. A. Wang W. Landrigan C. P.. (2011). Sleep disorders, health, and safety in police officers. JAMA 306:2567. 10.1001/jama.2011.1851, PMID: 22187276

[ref004] R Core Team. (2020). R: A language and environment for statistical computing. R Foundation for Statistical Computing, Vienna, Austria. Available at: https://www.R-project.org/

[ref46] Rohleder N. (2012). Acute and chronic stress induced changes in sensitivity of peripheral inflammatory pathways to the signals of multiple stress systems - 2011 Curt Richter award winner. Psychoneuroendocrinology 37, 307–316. 10.1016/j.psyneuen.2011.12.015, PMID: 22226321

[ref47] Rosenkranz M. A. Davidson R. J. MacCoon D. G. Sheridan J. F. Kalin N. H. Lutz A. (2013). A comparison of mindfulness-based stress reduction and an active control in modulation of neurogenic inflammation. Brain Behav. Immun. 27, 174–184. 10.1016/j.bbi.2012.10.013, PMID: 23092711 PMC3518553

[ref003] RStudio Team. (2020). RStudio: Integrated Development for R. RStudio, PBC, Boston, MA. Available at: http://www.rstudio.com/

[ref48] Saunders J. B. Aasland O. G. Babor T. F. De la Fuente J. R. Grant M. (1993). Development of the alocohol use disorders identification test (AUDIT): WHO collaborative project on early dection of persons with harmful alcohol consumption - II. Addiction 88, 791–804. 10.1111/j.1360-0443.1993.tb02093.x, PMID: 8329970

[ref49] Schlotz W. Hellhammer J. Schulz P. Stone A. A. (2004). Perceived work overload and chronic worrying predict weekend – weekday differences in the cortisol awakening response. Psychosom. Med. 66, 207–214. 10.1097/01.psy.0000116715.78238.56, PMID: 15039505

[ref50] Schmid C. Wiesli P. Bernays R. Bloch K. Zapf J. Zwimpfer C.. (2004). Decrease in sE-Selectin after pituitary surgery in patients with acromegaly. Clin. Chem. 50, 650–652. 10.1373/clinchem.2003.028779, PMID: 14981034

[ref51] Smith N. A. Voisin D. R. Yang J. P. Tung E. L. (2019). Keeping your guard up: hypervigilance among urban residents affected By community And police violence. Health Aff. 38, 1662–1669. 10.1377/hlthaff.2019.00560, PMID: 31589532 PMC7263347

[ref001] Stark S. M. Yassa M. A. Lacy J. W. Stark C. E. L. (2013). A task to assess behavioral pattern separation (BPS) in humans: Data from healthy aging and mild cognitive impairment. Neuropsychologia 51, 2442–2449. 10.1016/j.neuropsychologia.2012.12.014, PMID: 23313292 PMC3675184

[ref52] Strand K. J. Cutforth N. Stoecker R. Marullo S. Donohue P. (2003). Community-Based Research and Higher Education: Principles and Practices. San Francisco: Jossey-Bass.

[ref53] Syed S. Ashwick R. Schlosser M. Jones R. Rowe S. Billings J. (2020). Global prevalence and risk factors for mental health problems in police personnel: a systematic review and meta-analysis. Occup. Environ. Med. 77, 737–747. 10.1136/oemed-2020-106498, PMID: 32439827

[ref54] Thompson B. L. Waltz J. (2010). Mindfulness and experiential avoidance as predictors of posttraumatic stress disorder avoidance symptom severity. J. Anxiety Disord. 24, 409–415. 10.1016/j.janxdis.2010.02.005, PMID: 20304602

[ref55] Trombka M. Demarzo M. Campos D. Antonio S. B. (2021). Mindfulness training improves quality of life and reduces depression and anxiety symptoms among police officers: results From the POLICE study — a multicenter randomized controlled trial. Front. Psychiatry 12:624876. 10.3389/fpsyt.2021.624876, PMID: 33716824 PMC7952984

[ref56] Vago D. R. Silbersweig D. A. (2012). Self-awareness, self-regulation, and self-transcendence (S-ART): a framework for understanding the neurobiological mechanisms of mindfulness. Front. Hum. Neurosci. 6:296. 10.3389/fnhum.2012.00296, PMID: 23112770 PMC3480633

[ref57] Vickers A. J. Altman D. G. (2001). Analysing controlled trials with baseline and follow up measurements. Br. Med. J. 323, 1123–1124. 10.1136/bmj.323.7321.1123 11701584 PMC1121605

[ref58] Villalba D. K. Lindsay E. K. Marsland A. L. Greco C. M. Young S. Brown K. W.. (2019). Mindfulness training and systemic low-grade inflammation in stressed community adults: evidence from two randomized controlled trials. PLoS One 14:e0219120. 10.1371/journal.pone.0219120, PMID: 31295270 PMC6622480

[ref59] Violanti J. M. Charles L. E. McCanlies E. Hartley T. A. Baughman P. Andrew M. E.. (2017a). Police stressors and health: a state-of-the-art review. Policing 40, 642–656. 10.1108/PIJPSM-06-2016-0097, PMID: 30846905 PMC6400077

[ref60] Violanti J. M. Fekedulegn D. Andrew M. E. Hartley T. A. Charles L. E. Miller D. B.. (2017b). The impact of perceived intensity and frequency of police work occupational stressors on the cortisol awakening response (CAR): findings from the BCOPS study. Psychoneuroendocrinology 75, 124–131. 10.1016/j.psyneuen.2016.10.017, PMID: 27816820 PMC5135615

[ref61] Voigt R. Camp N. P. Prabhakaran V. Hamilton W. L. Hetey R. C. Griffiths C. M.. (2017). Language from police body camera footage shows racial disparities in officer respect. Proc. Natl. Acad. Sci. 114, 6521–6526. 10.1073/pnas.1702413114, PMID: 28584085 PMC5488942

[ref62] Wang X. Busch J. R. Banner J. Linnet K. Johansen S. S. (2019). Hair testing for cortisol by UPLC–MS/MS in a family: external cross-contamination from use of cortisol cream. Forensic Sci. Int. 305:109968. 10.1016/j.forsciint.2019.109968, PMID: 31622855

[ref63] Weathers F. W. Litz B. T. Keane T. M. Palmieri P. A. Marx B. P. Schnurr P. P. (2013). The PTSD Checklist for DSM-5 (PCL-5). National Center for PTSD, 5:2002.

[ref64] Weiss N. H. Tull M. T. Viana A. G. Anestis M. D. Gratz K. L. (2012). Impulsive behaviors as an emotion regulation strategy: examining associations between PTSD, emotion dysregulation, and impulsive behaviors among substance dependent inpatients. J. Anxiety Disord. 26, 453–458. 10.1016/j.janxdis.2012.01.007, PMID: 22366447 PMC3305816

[ref65] Wessa M. Rohleder N. Kirschbaum C. Flor H. (2006). Altered cortisol awakening response in posttraumatic stress disorder. Psychoneuroendocrinology 31, 209–215. 10.1016/j.psyneuen.2005.06.010, PMID: 16154709

[ref66] Wood G. Tyler T. R. Papachristos A. V. (2020). Procedural justice training reduces police use of force and complaints against officers. Proc. Natl. Acad. Sci. 117:201920671. 10.1073/pnas.1920671117, PMID: 32312803 PMC7211954

