# Supplementary material for: The Impact of Mindfulness Training on Police Officer Stress, Mental Health, and Salivary Cortisol Levels
Source: Front Psychol. 2021 Sep 3;12:720753. doi: 10.3389/fpsyg.2021.720753 (PMC8448191; doi:10.3389/fpsyg.2021.720753)
Supplement: Supplementary file 1 [file Data_Sheet_1.PDF]

Supplementary materials: The impact of mindfulness training on police officer stress, mental health, and salivary cortisol levels

Daniel W Grupe, Jonah L Stoller, Carmen Alonso, Chad McGehee, Chris Smith, Jeanette A Mumford, Melissa A Rosenkranz, and Richard J Davidson

Contents

2 Table S1: Final principal component analysis factor loading
3 Table S2: Differences between mindfulness training and waitlist control groups for additional psychological outcomes
4 Figure S1: Class engagement summary metrics
5 Figure S2: Null results for diurnal cortisol slope and hair cortisol concentration
6 Figure S3: Null results for inflammatory markers
7 Appendix 1: Curriculum overview
9 Appendix 2: Example practice log

Measure name	**Component 1** Distress/ Mental Health	**Component 2** Pain	**Component 3** Physical Health	**Component 4** Occupational Stress	**Component 5** Sleep Disturbances
PTSD Checklist	**0.67**	0.06	0.23	0.30	0.38
PROMIS Anxiety	**0.72**	0.03	0.06	0.42	-0.02
PROMIS Depression	**0.80**	0.08	0.09	0.18	0.10
PROMIS Fatigue	**0.75**	0.14	0.02	0.00	0.31
PROMIS Social Participation	**0.70**	0.00	0.08	-0.10	-0.04
Perceived Stress Scale	**0.71**	0.07	-0.03	0.25	0.30
OLBI-Exhaustion	**0.63**	0.03	-0.01	0.28	0.31
Work Limitations Questionnaire	**0.72**	-0.06	0.20	0.11	0.15
PROMIS Pain Intensity	0.03	**0.90**	0.09	0.09	0.14
PROMIS Pain Interference	0.09	**0.92**	0.08	0.07	0.05
Health Behavior Checklist	-0.27	0.04	**-0.82**	-0.03	0.02
PROMIS Physical Function	-0.02	0.23	**0.83**	0.10	0.12
Operational Police Stress Q.	0.34	0.05	0.09	**0.74**	0.25
Organizational Police Stress Q.	0.11	0.13	0.06	**0.92**	0.04
PROMIS Sleep Disturbances	0.24	0.12	-0.04	0.06	**0.90**
Pittsburgh Sleep Quality Inventory	0.22	0.09	0.15	0.16	**0.87**

Table S1: Final principal component analysis factor loading

Results of the final Principal Component analysis after removing scales that loaded < 0.40 on all 5 components (OLBI-Disengagement and Alcohol Use Disorders Identification Test. *Notes*: PTSD = Posttraumatic Stress Disorder; PROMIS = Patient-Reported Outcomes Measurement Information System; OLBI = Oldenburg Burnout Inventory.

	Omnibus Group*Time				Group Differences at Time 2					Group Differences at Time 3				
**Measure**	χ^2	p	p_{fdr}	η^2	t	df	p	p_{fdr}	d	t	df	p	p_{fdr}	d
**Five-Facet Mindfulness Q. – Short**	4.33	.11	-	.04	1.03	143.4	.30	-	.17	2.02	148.2	.05	-	.33
Observing One's Experience	9.06	.01	.05	.08	1.53	153.3	.13	.65	.25	3.01	158.4	.003	.02	.48
Describing One's Experience	.11	.95	.95	.00	.02	186.0	.99	.99	.00	.31	190.1	.76	.77	.05
Acting with Awareness	3.04	.22	.55	.03	.60	158.8	.55	.98	.10	1.68	163.7	.10	.25	.26
Non-Judging of Inner Experience	.51	.78	.95	.00	.53	157.1	.59	.98	.09	.70	162.1	.49	.77	.11
Non-Reactivity to Inner Experience	.30	.86	.95	.00	-.16	151.2	.88	.99	-.03	.30	156.2	.77	.77	.05
**Brief Resilience Scale**	8.30	.02	-	.07	2.45	194.4	.02	-	.35	2.30	196.7	.02	-	.33
**Emotional Style Questionnaire[b]**	-	-	-	-	-	-	-	-	-	-	-	-	-	-
Outlook	1.63	.44	.80	.03	1.28	77.4	.20	.54	.30	.70	79.7	.49	.73	.16
Resilience	.59	.75	.82	.01	.55	86.9	.59	.61	.12	.71	88.8	.48	.73	.15
Social Intuition	2.54	.28	.80	.05	.77	74.0	.44	.61	.18	1.58	76.2	.12	.36	.37
Self-Awareness	1.27	.53	.80	.02	1.11	91.9	.27	.54	.23	.51	92.9	.61	.73	.11
Sensitivity to Context	5.41	.07	.42	.11	1.93	67.9	.06	.36	.47	2.35	68.9	.02	.12	.57
Attention	.39	.82	.82	.01	0.51	95.9	.61	.61	.10	-.20	96.8	.85	.85	-.04
**Work-Family Spillover**	-	-	-	-	-	-	-	-	-	-	-	-	-	-
Negative Work-to-Family Spillover	.33	.85	.90	.00	-.57	190.6	.57	.70	-.08	-.11	193.6	.91	.91	-.02
Positive Work-to-Family Spillover	1.60	.45	.90	.01	-.57	195.1	.57	.70	-.08	.90	197.5	.37	.74	.13
Negative Family-to-Work Spillover	.21	.90	.90	.00	-.38	203.9	.70	.70	-.05	.15	205.6	.88	.91	.02
Positive Family-to-Work Spillover	2.65	.27	.90	.02	-.38	181.0	.70	.70	-.06	-1.59	183.4	.11	.44	-.24

Table S2. Differences between mindfulness training and waitlist control groups for additional psychological outcomes

Notes: Statistics are the results of linear mixed effects models adjusted for baseline scores with covariates of gender, years of police experience, and cohort (year 1/year 2) and a random intercept for each participant. p_{fdr} = false discovery rate-corrected *p* values for subscales of each measure. **a.** For the Five-Facet Mindfulness Questionnaire, an error in survey administration resulted in items 19-24 not being presented at baseline for participants in Cohort 1. This included one item each from "observing one's experience" and "non-reactivity", and two items each in "acting with awareness" and "non-judgment". This mistake was identified and corrected before subsequent assessments. Rather than abandoning data for Cohort 1, we imputed missing data for each subscale using the mean score of items collected for that subscale. Independent sample t-test results indicated that means of the extrapolated subscales for Cohort 1 were not different from the corresponding subscales for Cohort 2, *ts* < .8, *ps* > .4. **b.** The Emotional Style Questionnaire was only obtained from participants in Cohort 2.

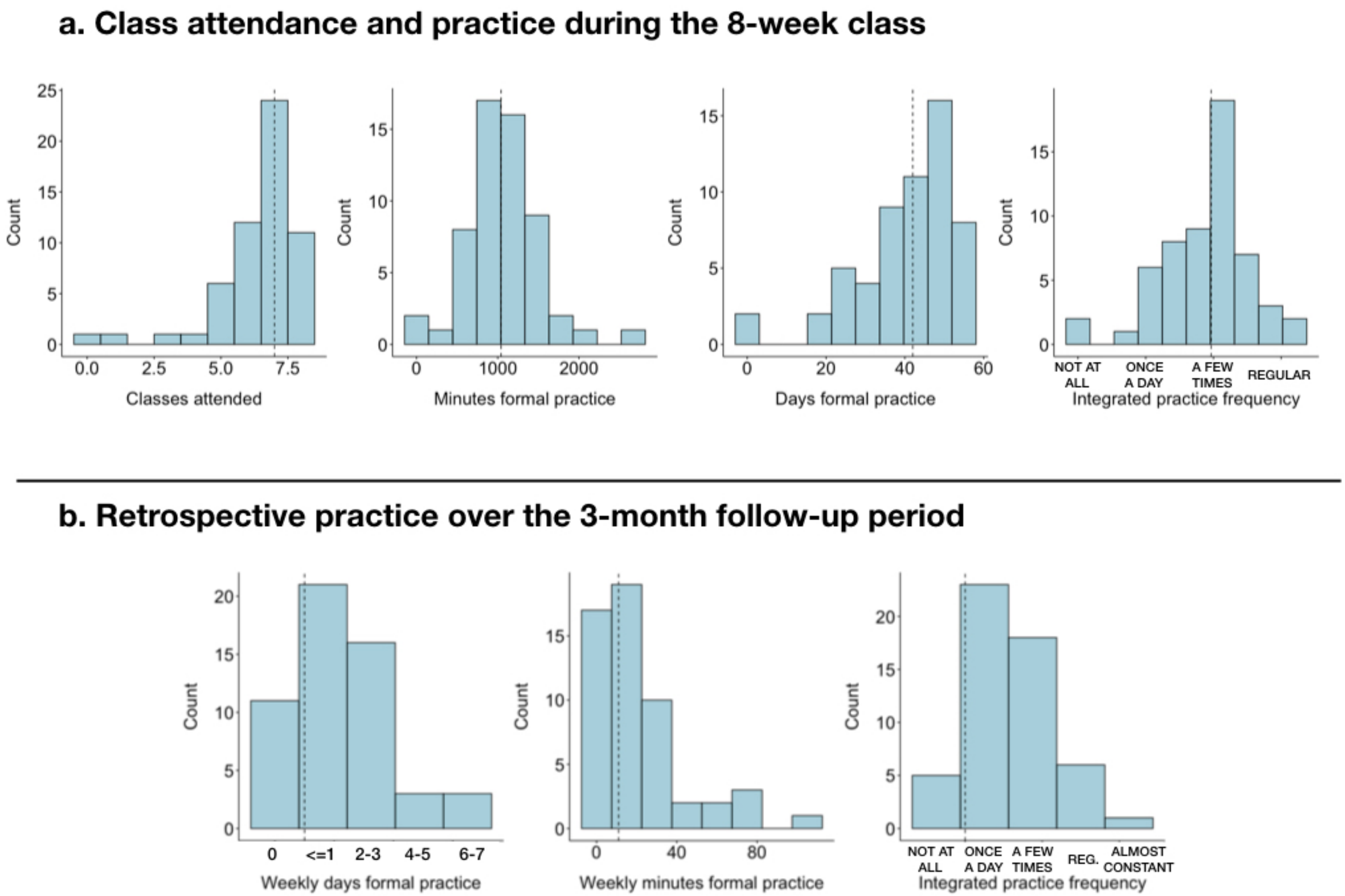

Figure S1. Class engagement summary metrics.
a. Metrics of engagement during the 8-week class included class attendance and 3 outcomes from weekly practice logs: Total minutes of formal practice, total days of formal practice, and typical daily frequency of informal/integrated practice. **b.** Metrics of engagement between the end of the class and the 3-month follow-up assessment were obtained from single questions, at the follow-up assessment, asking about weekly days of formal practice, average practice duration (which was multiplied by weekly frequency to generate an estimated weekly minutes metric), and typical daily frequency of informal/integrated practice.

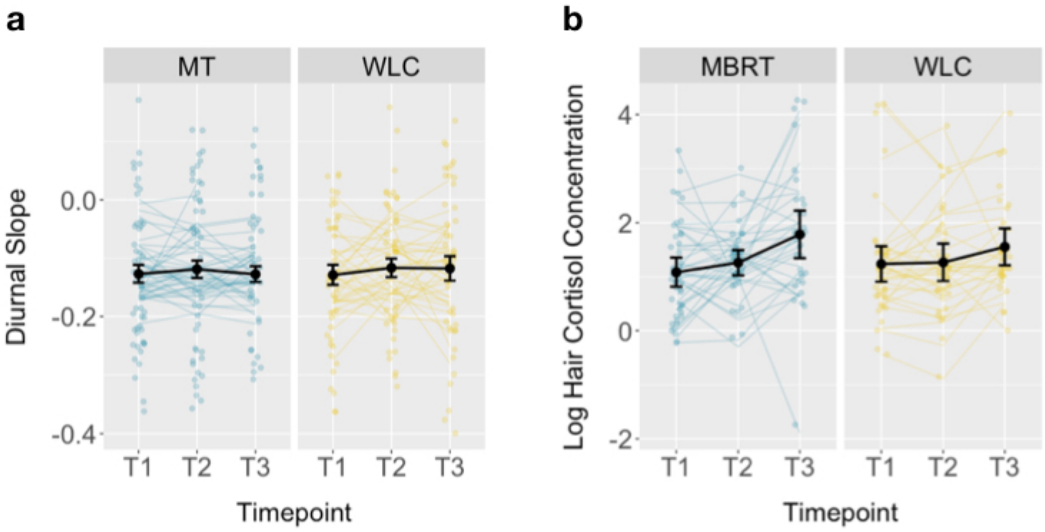

Figure S2. Null results for diurnal cortisol slope and hair cortisol concentration
a. Log-transformed diurnal cortisol slope for the mindfulness training (MT) and waitlist control (WLC) groups at baseline (T1), post-mindfulness training (T2), and 3-month follow-up (T3). Controlling for T1 slope, the two groups did not differ at either T2 or T3 ($|t\text{s}| < 1.0$, $p\text{s} > 0.3$). **b.** Log-transformed hair cortisol concentration for the mindfulness training (MT) and waitlist control (WLC) groups at baseline (T1), post-mindfulness training (T2), and 3-month follow-up (T3). Controlling for T1 hair cortisol concentration, the two groups did not differ at either T2 or T3 ($|t\text{s}| < 1.1$, $p\text{s} > 0.3$).

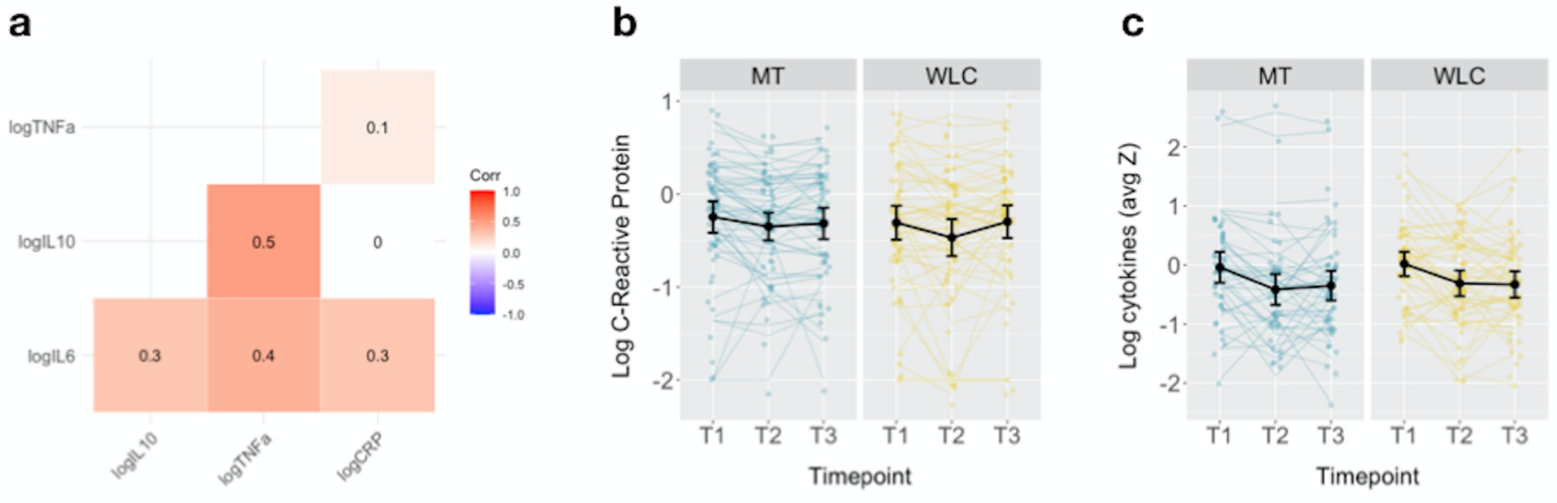

Figure S3. Null results for inflammatory markers
a. Correlation matrix for log-transformed baseline values of inflammatory markers shows moderately high correlations between the inflammatory cytokines interleukin IL-6, IL-10, and TNF-alpha, but generally weak correlations between C-reactive protein (CRP) and these cytokines.
b. Log-transformed C-reactive protein for the mindfulness training (MT) and waitlist control (WLC) groups at baseline (T1), post-mindfulness training (T2), and 3-month follow-up (T3). Controlling for T1 CRP levels, the two groups did not differ at either T2 or T3 ($|ts| < 0.9$, $ps > 0.3$). **c.** Log-transformed average of Z-transformed cytokine 3-plex for the mindfulness training (MT) and waitlist control (WLC) groups at baseline (T1), post-mindfulness training (T2), and 3-month follow-up (T3). Controlling for T1 cytokine levels, the two groups did not differ at either T2 or T3 ($|ts| < 0.5$, $ps > 0.6$).

Appendix 1: Intervention Summary

Overview of 8-week mindfulness curriculum, adapted from our previous work (Grupe et al., 2021) and inspired by the Mindfulness-Based Resilience Training curriculum created by Mike Christopher, Rich Goerling, and Brant Rogers at Pacific University (Christopher et al., 2016, 2018). We are deeply grateful to them for sharing their wisdom and experience with us.

Week 1: Introduction to Resilience and Mindfulness

Content: Introduction of course. Definitions of mindfulness, neuroplasticity and how we can foster greater resilience. Creation of community agreements and discussion of culture. Movement and body scan practices. Debriefing of session 1 practices.

Homework: Explanation of practice log. Formal practice: 9 minutes movement or 9 minutes body scan 6 out of 7 days. Informal practice: "Dropping in" and 3 good breaths.

Week 2: Settling into Resilience and Mindfulness

Content: Normalizing mind wandering and addressing other (mis)conceptions about doing the practices "correctly." Movement, body scan and debrief. Introduce walking practice.

Homework: Formal practice: Alternate 9 minutes movement and 9 minutes body scan 6 out of 7 days. 5 minutes of walking practice 3 times. Informal practice: "Dropping in" and 3 good breaths.

Week 3: Meeting our experiences through Resilience and Mindfulness

Content: Movement, exploration of physical posture in sitting meditation, introduction to breath awareness practice, and debrief. Reading and video to reconnect to participants' inspiration and motivation.

Homework: Formal practice: Alternate 9 minutes movement and 5 minutes breath awareness practice 6 out of 7 days. Informal practice: Pleasant Events Calendar, integrated walking practice and 3 good breaths.

Week 4: Meeting Stress with Resilience and Mindfulness

Content: Review and discuss Pleasant Events Calendar. Movement practice, breath awareness practice, and debrief. Introduction of mindful eating.

Homework: Formal practice: Alternate 15 minutes movement and 15 minutes breath awareness practice. Informal practice: Integrated walking practice, Unpleasant Events Calendar and 3 good breaths.

Week 5: Reactivity, Resilience and Mindfulness

Content: Review and discuss Unpleasant Events Calendar. Movement practice, breath awareness practice, and debrief. Reflection of practice and course at halfway point and resetting of intentions for the remaining 4 weeks. Introduction to compassion practice.

Homework: Alternate movement/breath awareness practice with compassion practice 6 days out of 7.
Informal practice: Walking practice (1x per week) and 3 good breaths.

Week 6: Compassion, Resilience and Mindfulness

Content: Movement, awareness of breath practice, compassion practices, and debriefing. Discussion and preparation for the day of extended practice.

Homework: Alternate between 15 minutes breath awareness and compassion practice.
Informal practice: Walking practice (1x per week), and 3 good breaths.

Week 7: Extended Practice Session (4 hours)

Content: This session includes all of the previous practices along with mountain meditation and a period of silent mindful practice.

Homework: Practice log. Participant decides their practice.

Week 8: Resilience and Mindfulness: Beginning Again

Content: Movement practice, breath awareness practice, and debriefing. Debriefing about the course. Review of resources and supports for participants. Plan for implementation and integration of mindfulness after the class.

Homework: Practice Log. Participant decides their practice.

References

Christopher, M. S., Goerling, R. J., Rogers, B. S., Hunsinger, M., Baron, G., Bergman, A. L., & Zava, D. T. (2016). A Pilot Study Evaluating the Effectiveness of a Mindfulness-Based Intervention on Cortisol Awakening Response and Health Outcomes among Law Enforcement Officers. *Journal of Police and Criminal Psychology*, *31*(1), 15–28. https://doi.org/10.1007/s11896-015-9161-x

Christopher, M. S., Hunsinger, M., Goerling, R. J., Bowen, S., Rogers, B. S., Gross, C. R., … Pruessner, J. C. (2018). Mindfulness-based resilience training to reduce health risk, stress reactivity, and aggression among law enforcement officers: A feasibility and preliminary efficacy trial. *Psychiatry Research*, *264*, 104–115. https://doi.org/10.1016/j.psychres.2018.03.059

Grupe, D. W., McGehee, C., Smith, C., Francis, A. D., Mumford, J. A., & Davidson, R. J. (2021). Mindfulness Training Reduces PTSD Symptoms and Improves Stress-Related Health Outcomes in Police Officers. *Journal of Police and Criminal Psychology*, *36*, 72–85. https://doi.org/10.1007/s11896-019-09351-4

	FORMAL PRACTICE	INTEGRATED PRACTICE	NOTES AND OBSERVATIONS
M	☐ Body Scan _______ mins<br>☐ Sitting _______ mins<br>☐ Walking _______ mins<br>☐ Movement _______ mins<br>☐ Compassion _______ mins<br>☐ Other _______ mins	☐ Not at all<br>☐ Once<br>☐ A few times<br>☐ Regularly<br>☐ Almost constantly	
T	☐ Body Scan _______ mins<br>☐ Sitting _______ mins<br>☐ Walking _______ mins<br>☐ Movement _______ mins<br>☐ Compassion _______ mins<br>☐ Other _______ mins	☐ Not at all<br>☐ Once<br>☐ A few times<br>☐ Regularly<br>☐ Almost constantly	
W	☐ Body Scan _______ mins<br>☐ Sitting _______ mins<br>☐ Walking _______ mins<br>☐ Movement _______ mins<br>☐ Compassion _______ mins<br>☐ Other _______ mins	☐ Not at all<br>☐ Once<br>☐ A few times<br>☐ Regularly<br>☐ Almost constantly	
TH	☐ Body Scan _______ mins<br>☐ Sitting _______ mins<br>☐ Walking _______ mins<br>☐ Movement _______ mins<br>☐ Compassion _______ mins<br>☐ Other _______ mins	☐ Not at all<br>☐ Once<br>☐ A few times<br>☐ Regularly<br>☐ Almost constantly	
F	☐ Body Scan _______ mins<br>☐ Sitting _______ mins<br>☐ Walking _______ mins<br>☐ Movement _______ mins<br>☐ Compassion _______ mins<br>☐ Other _______ mins	☐ Not at all<br>☐ Once<br>☐ A few times<br>☐ Regularly<br>☐ Almost constantly	
SA	☐ Body Scan _______ mins<br>☐ Sitting _______ mins<br>☐ Walking _______ mins<br>☐ Movement _______ mins<br>☐ Compassion _______ mins<br>☐ Other _______ mins	☐ Not at all<br>☐ Once<br>☐ A few times<br>☐ Regularly<br>☐ Almost constantly	
SU	☐ Body Scan _______ mins<br>☐ Sitting _______ mins<br>☐ Walking _______ mins<br>☐ Movement _______ mins<br>☐ Compassion _______ mins<br>☐ Other _______ mins	☐ Not at all<br>☐ Once<br>☐ A few times<br>☐ Regularly<br>☐ Almost constantly	

FORMAL PRACTICE
Check the type or types of formal practice you have done for the day and record the number of minutes you practiced. **Do not include time spent practicing in class in your practice log.** We will keep track of this time separately.

INTEGRATED PRACTICE
How often did you take part in integrated practice, or moments of mindful awareness, during the day? (for example, paying attention to the sensation of breathing, bringing awareness to your posture or body sensations, walking mindfully, mindful conversations, etc.)

NOTES AND OBSERVATIONS
Record any insights, observations, challenges, or questions that come up related to the practices during the week.
